# Evaluation of Slag Reaction Efficiency in Slag-Cement Mortars under Different Curing Temperature

**DOI:** 10.3390/ma12182875

**Published:** 2019-09-05

**Authors:** Liang Wang, Hongzhu Quan, Qiuyi Li

**Affiliations:** College of Civil Engineering & Architecture, Qingdao Agricultural University, Qingdao 266109, China (L.W.) (Q.L.)

**Keywords:** blast furnace slag, compressive strength, calcium hydroxide, effective replacement ratio, reaction efficiency coefficient

## Abstract

At present, not many studies have considered methods to quantitatively evaluate the reaction efficiency of granulated blast furnace slag (GBFS) at different curing temperatures. For high volume slag concrete, when the replacement ratio exceeds a certain ’threshold’ value, the superfluous and ineffective slag will no longer react in concrete but simply behave as a fine aggregate, which may cause the decrement of strength. The ’threshold’ value depends on the reaction efficiency of slag. In this study, experiments on mortars with different replacement ratios by slag were conducted at different curing temperatures (20, 30, and 50 °C, respectively), the threshold values of effective replacement ratio by slag were comprehensively analyzed through the reaction efficiency of slag mortar. The results showed that the turning point of the strength curve with replacement ratio can be considered as the threshold value of the effective replacement ratio by slag in mortar. Along with the curing temperature enhancement, the threshold value of the effective replacement ratio by slag in concrete decreased, whereas the reaction efficiency of slag increased. Meanwhile, the analysis of cement effective coefficient (k value) and basicity was also calculated. Based on the obtained threshold values of effective replacement ratio at different curing temperatures, the formula for the determination of reaction efficiency coefficient of slag in the mortar can be established. Therefore, the reaction efficiency coefficient and upper limit of the effective replacement ratio of slag at different temperatures can be calculated more intuitively and quantitatively, providing a theoretical basis and reference for practical engineering applications.

## 1. Introduction

According to the Market Development Research and Investment Prospects Report of Slag Powder Industry in China, the output of the slag powder industry in 2018 is huge, at 103 million tons [1]. However, due to the development slowdown of infrastructure construction and real estate industry, the overall downturn of the building materials industry has brought about higher requirements for the utilization of slag powder in practical engineering applications, with the use of high content granulated blast furnace slag (GBFS) not allowed in more demanding environments, the proportion of slag powder also would be reduced further, and the comprehensive utilization of slag powder is facing serious challenges [2]. Therefore, on the basis of guaranteeing project quality, how to determine the upper limit of effective replacement ratio of slag powder in high volume slag concrete and achieve the high efficiency utilization of slag has become very important.

Ishida T reported that with the increase of the replacement ratio by granulated blast furnace slag (GBFS), the Ca(OH)_2_ amount of concrete decreased, and the consumed Ca(OH)_2_ amount increased because of the slag hydration. When the replacement ratio was over 67%, the remaining Ca(OH)_2_ amount was close to 0 [3]. Thus it may be known, when the replacement ratio exceeds a certain ’threshold’ value, Ca(OH)_2_ will be almost completely consumed, and the redundant and ineffective slag in concrete would no longer react with Ca(OH)_2_, but just behaves as fine aggregate, leading to a decrease in strength. Therefore, the so-called ’threshold’ value may be considered as the upper limit of the effective replacement ratio. Many researchers have studied the upper limit of the replacement ratio of slag at ambient temperature [4,5,6,7,8,9].It can be concluded that the hydration was slowed down when the addition of GBFS went up to 50–70%, the upper limit of the replacement ratio should not exceed 70% due to the reduction of strength. However, curing temperature has a big influence on the “threshold” value of the replacement ratio due to the reactivity of GBFS at different temperatures. Hosokawa D. reported that at the replacement ratio of 70%, no matter what the curing age was, the higher the curing temperature, the larger the compressive strength of slag concrete. The strength of slag concrete at 56 days at the curing temperature of 40 °C increased by 30% more than that of 20 °C [10]. Saio T reported that in the case of a curing temperature of 40 and 60 °C, the heat of hydration and the combined water amount became greater, and the reaction activity of slag was higher than that of 20 °C [11]. Nagao Y. proved under the conditions of different curing temperatures the strength development curve was different due to the reaction activity [12]. Bougara A. discussed that the greatest benefit in terms of strength and hydration are achieved depending on slag reactivity, this reactivity is due to the chemical composition and curing temperature of the slag [13]. Huseien G.F. revealed that the reaction products and the strengths of slag mortar depended strongly on the nature of compositions and curing temperatures [14]. Ogirigbo O.R. showed that curing temperature had a much greater influence on the reactivity of the slags than the difference in chemical composition, the higher temperature resulted in an increase in the degree of hydration of the slags [15]. In practical engineering construction, the reaction efficiency of slag at different ambient temperatures will directly affect the effective replacement ratio in different seasons. Therefore, it is necessary to study the relationship between reaction efficiency of slag and temperature. At present, there are very limited studies available on the quantitative evaluation methods for the reaction efficiency of slag at different curing temperatures. How to calculate the reaction efficiency coefficient of GBFS is of great significance for determining the threshold value of effective replacement ratio of GBFS at different temperatures. The present research is aimed to bridge the aforementioned gap.

Generally speaking, cracks will occur in high volume slag concrete due to temperature rise caused by internal hydration heat [16], the adiabatic temperature rise characteristic of mortar or concrete can be used to predict the temperature change and temperature cracking index in the mortar and concrete [17]. In this study, in order to ensure the volume stability and durability of high-volume slag concrete, based upon the calculation of the adiabatic temperature rise of BB (blast furnace slag) cement, the final adiabatic temperature rise is close to 50 °C. Therefore, the curing temperatures in this study were set to 20, 30 and 50 °C, respectively. Through the way of thermal curing, the relationship between the replacement ratio of slag and the properties of mortar under three curing temperatures (20, 30 and 50 °C) is analyzed, the threshold values of the effective replacement ratio of slag at different curing temperatures were determined eventually. According to the results of cement effective coefficient (k value) and basicity, the reaction efficiency of slag at different curing temperatures can be analyzed qualitatively, and the calculation formula for the reaction efficiency coefficient is put forward. On this basis, the relationship Equation between the curing temperature and reaction efficiency coefficient is established. According to the relationship Equation, the effective replacement ratio of slag at different temperatures can be calculated directly. In addition, because the reaction efficiency of slag varies with curing methods at the same temperature, conversion formulae of the reaction efficiency coefficient under different curing methods are also proposed. The results can provide a theoretical basis for the practical construction of high-volume slag concrete under different temperature conditions and have a certain significance.

## 2. Experimental

### 2.1. Materials and Mix Proportions

**Figure 1:**
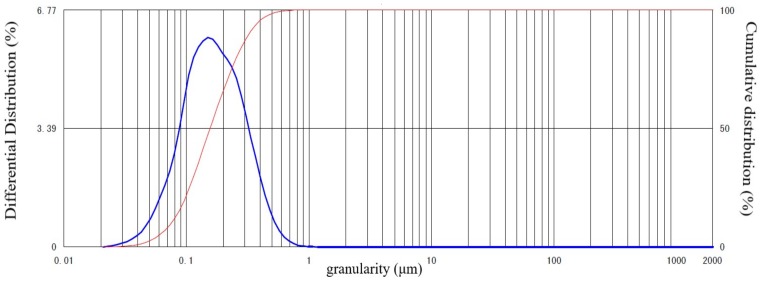
The granularity distribution of blast furnace slag used in this study.

**Figure 2:**
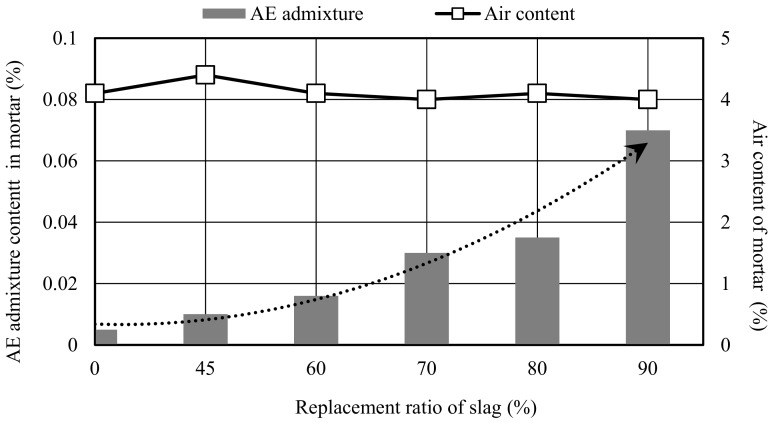
Air entraining admixture (AE) admixture content and air content of different slag mortar.

Ordinary Portland cement that met the requirements of Chinese National Standard (GB/T175-2007), “Portland Cement (Shanshui Cement Co., Ltd., Qingdao, China)” was used in this study. The physical properties of cement are shown in Table 1. Blast furnace slag which met the requirements of Chinese National Standard (GB/T203-2008) was used. The density and the specific surface area of the slag were 2.88 g/cm^3^ and 3830 cm^2^/g, respectively. The chemical composition is shown in Table 2, the particle size distribution is shown in Figure 1. The China ISO standard sand for mortar specified in Chinese National (GB/T 14684-2011) was used as fine aggregate (Xiamen ISO Standard Sand Co., Ltd, Xiamen, China), the particle size ranges were 0.08–2 mm, the content of SiO2 was 99.2%, the water absorption was 0.01%, and the moisture content was 0.02%. In order to guarantee a good workability and durability of mortar or concrete, reducing bleeding, settlement and segregation of mortar, an air entraining admixture (AE, Sobute New Materials Co., Ltd., Nanjing, China) with a water-reduction effect was diluted 100 times in accordance with Chinese National Standard for Chemical Admixtures for Concrete and was used to control the flow values and air contents of different slag mortars in the same levels, and the target air content was designed as 4 ± 0.5% and flow value was designed as 20 ± 1 cm. The air entraining admixture can reduce the water consumption of concrete by 8–10%, and thus compensate for the strength decline caused by the increase of porosity. Through several table flow testing experiments for different slag mortars, the required amounts of AE admixture for different slag mortars were determined. The relationships between the AE admixtures and replacement ratio are shown in Figure 2. Table 3 presents the detailed mix proportions of slag mortar. The volume ratio of paste and sand in the mortar was determined as 1: 1 in order to emphasize the changes of paste. The water to binder ratio (W/(SL + C)) of 0.5 was used to prepare specimens throughout the experimental program, and the replacement ratios of slag in the mortar were designed as 0, 40, 50, 60, 70, 80 and 90%, respectively. In addition, the water to cement ratios of 0.4 and 0.6 were also designed in order to compare and calculate the cement effective coefficient (k value) [18].

### 2.2. Mortar Preparation and Curing

Mortar specimens in the shape of Φ50 mm×100 mm cylinders (Huayun Experimental Instruments Co., Ltd., Cangzhou, China) were prepared for testing. To make sure that the preparation temperature was close to the target curing temperature, the temperature of the mixing water during the preparation was controlled at 20, 30 and 50 °C, respectively. The cement and sand were also placed in a constant temperature equipment (Liuqin Testing Instrument Co., Ltd., Dongguan, China) to reach the required temperature (20, 30, and 50 °C) for preparation at the same time. Although there were some certain errors between the preparation temperature and target temperature (about ± 1 °C) from the beginning to the end of preparation due to heat dissipation, the impact was not serious. After the demolding of the mortar, it was kept at specified temperature conditions to start the curing. Regarding the curing conditions, after demolding the mortar for one day, the mortar specimens were placed into a sealing bag half-filled with water so that the specimens are completely immersed in the water. Then, the sealed water bag with the specimens was placed in constant temperature equipment to start the sealed curing under conditions of 20, 30, and 50 °C, respectively. The constant temperature equipment was opened every 3 days to check the content of water in bag to ensure the specimens were completely immersed in the water.

### 2.3. Experimental Method

Based on GB/T 50081-2002, the specimens with in the shape of Φ50 × 100 mm cylinders were prepared for the tests of compressive strength of different slag mortars, the tests were conducted at the ages of 3, 7, 14, 28, and 91 days, respectively, three identical specimens were tested per age for each specimen design. The differential thermal gravimetry analysis (TG/DTA) method was used in this study to measure the existing and consumed amount of Ca(OH)_2_. The detailed process method of samples was as follows. The center part of mortar specimen was cut off by a cutting machine (Shengxing Instruments Equipment Co., Ltd., Cangzhou, China), a small slice with diameter 50 mm and height 5 mm was obtained, then broken into 2.5–5.0 mm small particles with a long flat nose pliers, the hydration reaction of specimen particle has stopped after soaking in acetone for 24 hours, and the particles were put in a vacuum chamber for drying and preservation after evaporation of acetone. Then the particles were ground into powder by contusion mortar, the powder through 40 μm sieve has been collected as the samples for the test. The amount of Ca(OH)_2_ in mortar was generated approximately at 450–500 °C by dehydroxylation. The range of testing temperature was from room temperature to 1000 °C. The rate of temperature rise was 20 °C /min [19].

## 3. Results and Discussion

### 3.1. Compressive Strength Development

**Figure 3:**
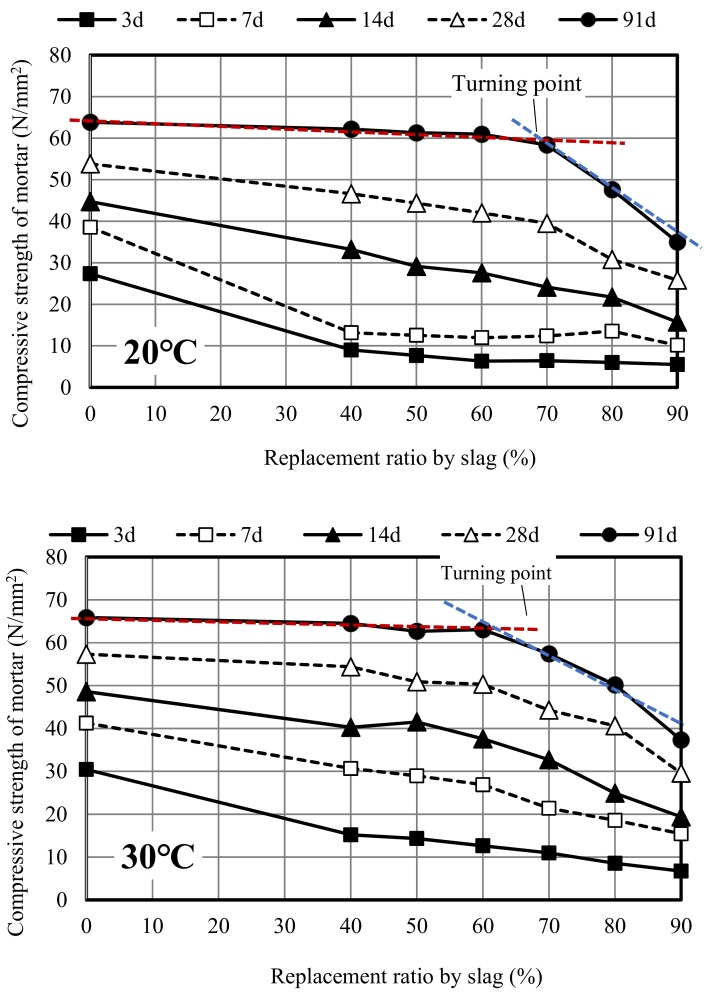

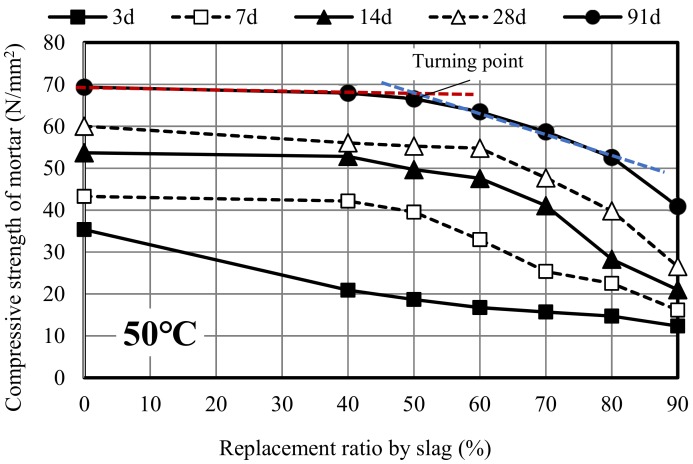
Compressive strengths of slag mortar at different curing temperatures.

Figure 3 illustrates the relationship between the replacement ratio by the slag and compressive strength of mortar at different curing ages under different curing temperatures. It can be seen that at a curing temperature of 20 °C and at an early age, the strength of mortar obviously decreases with the increase of the replacement ratio, nearly showing a linear decline correlation. This is probably because the strength of the slag alone is lower than that of ordinary cement, and the reaction of slag in mortar was not activated yet. However, the strength curve of mortar at 91 days still decreases with the overall replacement ratio, but its decline slows down significantly and there is an obvious turning point on the strength curve which occurs at the replacement ratio of 70%. When the replacement ratio was lower than 70%, the strength of the mortar decreased slowly and had a liner relationship with the replacement ratio. In contrast, the strength became very low and dropped sharply when replacement ratio exceeded 70%. This is consistent with the results of other researchers, which compared the ordinary cement concrete with the 70% replacement ratio slag concrete, a similar strength level could be obtained without significant loss [20,21]. This is likely to be because the effective slag in mortar at this time has been almost completely consumed and the superfluous slag in mortar no longer played a role but just filled in the mortar as a fine aggregate similar to sand, leading to the increase of actual water/binder ratio and the sharp decrease in strength. Therefore, the replacement ratio at the turning point of the strength curve could represent the threshold value of effective replacement ratio by the slag in mortar. In addition, although the reaction of slag in mortar was further activated at 91 days, the gaps between the strengths of ordinary mortar and different slag mortars were further narrowed, which to a certain extent could make up for the strength loss due to the slag in mortar, the strength of slag mortar was still lower than that of ordinary mortar. This also agreed with the result of Abdelli K. who believed the GBFS reacts very late at room temperature due to low activity, the strength of GBFS concrete was worse than ordinary concrete [22].

At the curing temperature of 30 °C, unlike the results at 20 °C, the turning point of the strength curve moved to a lower replacement ratio and occurred at the replacement ratio of 60%. The strength showed a similar or higher value compared with ordinary mortar under high temperature curing at the age of 91 days. These results revealed that a high curing temperature can accelerate the hydration reaction and activate the reaction of slag [23]; therefore, the strength of slag mortar can reach the same level as ordinary cement mortar and fit a straight line better with a replacement ratio when the replacement ratio was lower than 60%. At more than 60%, the strengths of slag mortar decreased obviously. At the curing temperature of 50 °C, the higher temperature can further accelerate the hydration reaction of mortar. At 91 days, the turning point of the strength curve moved further forward and occurred at the replacement ratio of 50%. When the replacement ratio of slag exceeded 50%, the strength decreased with replacement ratio; however, there was no significant loss in strength at the replacement ratio of 50%.

In general, many researchers believed that the threshold value of the effective replacement ratio by slag in mortar would increase with the curing temperature [15,24]. In other words, the higher the curing temperature, the more the amount of slag involved in the reaction. However, based on the results discussed above, a contrary conclusion has been obtained.

### 3.2. Relationship Between Curing Temperature and Compressive Strength

**Figure 4:**
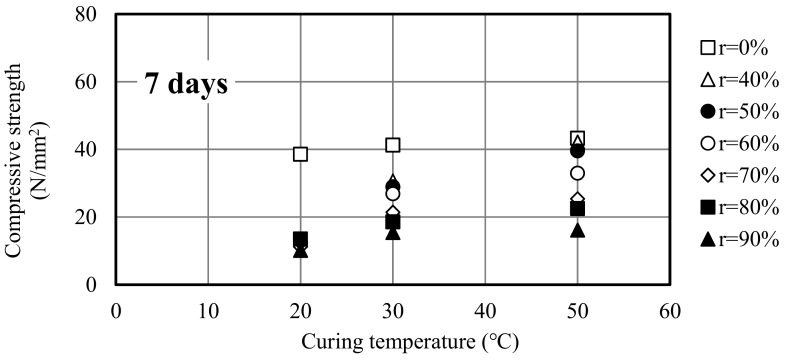

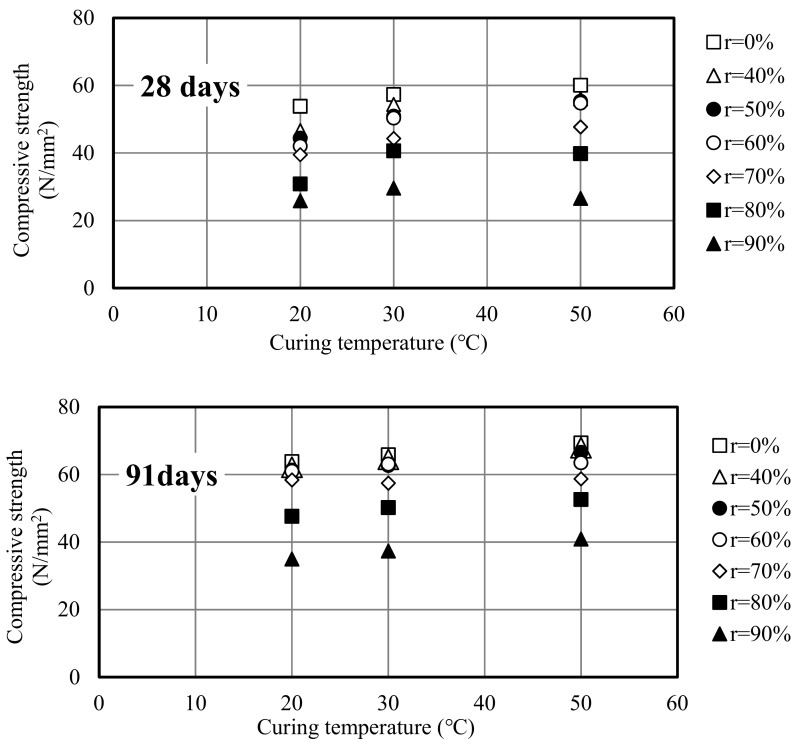
Relationship between curing temperature and compressive strength at different curing ages.

Figure 4 illustrates the relationship between the curing temperature and compressive strength of different slag mortars. When the replacement ratio remained the same, the strength of slag mortar increased with the increasing curing temperature. A high curing temperature can accelerate the hydration reaction of cement in the mortar to produce more C-S-H gels and Ca(OH)_2_, and the reaction can be further stimulated to react with the hydration production to improve the strength of mortar. This showed that the dependence of slag mortar on curing temperature was high. Huang H. reported that the addition of slag was unfavorable for the structural build-up of cement paste at 10 and 20 °C, however, a positive effect was observed at high temperature of 40 °C [25]. The higher the curing temperature, the more significant the role of slag in mortar, and the reaction efficiency of slag was higher. However, when the replacement ratio is too high, the effect of the curing temperature was not significant.

### 3.3. Changes in Ca(OH)_2_ Amount with Replacement Ratio by Slag

**Figure 5:**
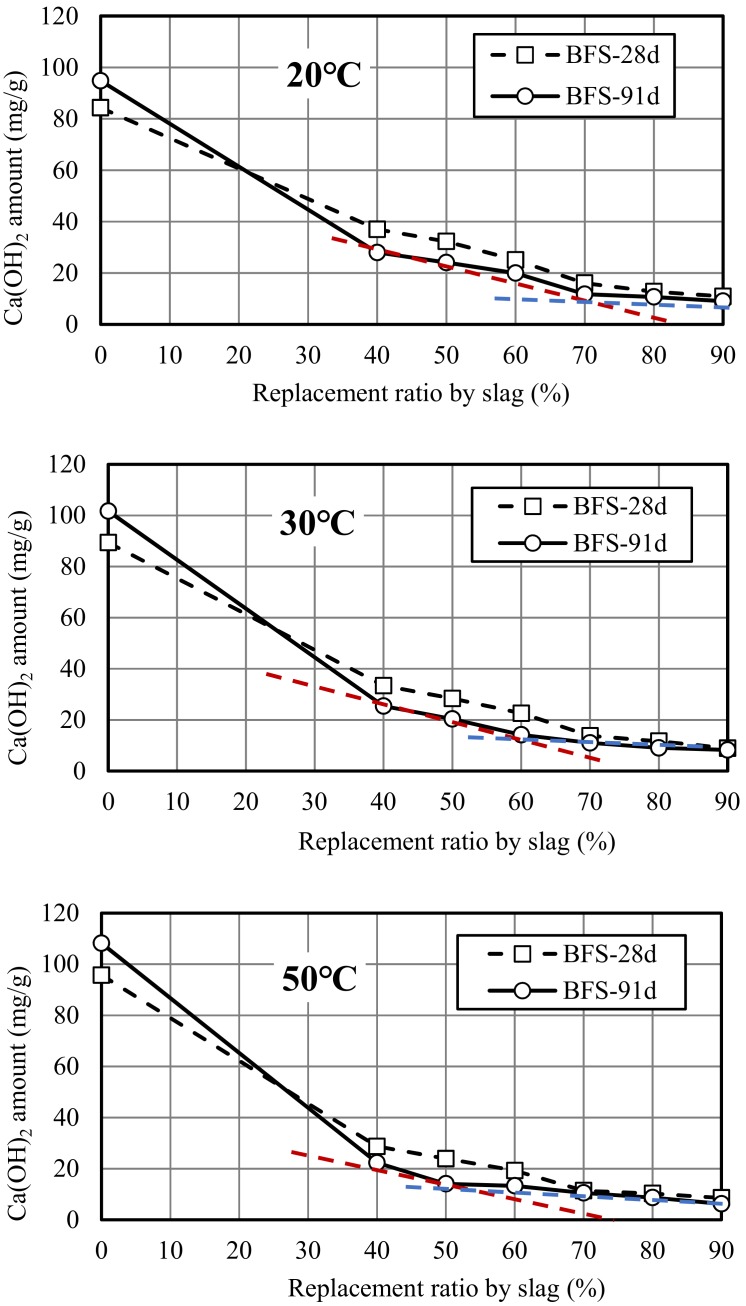
Amount of Ca(OH)_2_ in slag mortar at different curing temperatures.

Figure 5 illustrates the relationship between the amount of Ca(OH)_2_ and replacement ratio by slag at different curing temperatures. Firstly, at 20 °C, because the reaction is not significant, the contribution of slag to the consumption of Ca(OH)_2_ was relatively low than those at high temperatures, the amount of Ca(OH)_2_ at 28 days decreased linearly with the replacement ratio. However, there were two obvious turning points for the change curve of the amount of Ca(OH)_2_ at 91 days, which occurred at the replacement ratio of 40% and 70%. For the first turning point, compared with ordinary Portland cement mortar, Ca(OH)_2_ in the mortar started to be consumed by slag at the replacement ratio of 40%, resulting in a sharp decrease of the amount of Ca(OH)_2_. When replacement ratio of slag was 70%, the amount of Ca(OH)_2_ produced by the remaining 30% cement in the mortar at 91 days could be consumed to the lowest value or the so-called threshold value. When the replacement ratio exceeded 70%, the amount of Ca(OH)_2_ produced by the cement was insufficient, the superfluous slag no longer played a role in consuming Ca(OH)_2_, therefore, the amount of Ca(OH)_2_ almost unchanged with the change in replacement ratio. At 30 °C curing temperature, unlike the result at 20 °C curing temperature, the second turning point of the amount of Ca(OH)_2_ curve at 91 days moved to a lower replacement ratio of 60%. This showed that the high curing temperature also had an influence on the turning point of the change curve of the amount of Ca(OH)_2_. At the replacement ratio of 60%, the Ca(OH)_2_ amount produced by the remaining 40% cement in mortar could exactly be consumed to the lowest value by 60% slag. At more than 60%, the change curve of the amount of Ca(OH)_2_ in mortar was basically close to a straight line. In contrast, at 50 °C curing temperature, the turning point of the Ca(OH)_2_ curve moved further forward at the replacement ratio of 50%. Combined with the results of strength curves, it can be said that 70%, 60%, and 50% are the threshold values of effective replacement ratio of slag at 20, 30, and 50°C. Kokubu K reported that under the condition of same replacement ratio, with the increase of curing temperature, the residual amount of Ca(OH)_2_ in concrete decreased, the residual amount of Ca(OH)_2_ at a high temperature reached the lowest value faster [26].

According to Equation (1), the Ca(OH)_2_ consumption by 1 g slag could be calculated.

(1) $$CH_{A}=\frac{\left(CH_{PC}\times \left(1-r\right)-CH_{SL}\right)}{r}$$

where:

*CHA*: Consumption of Ca(OH)_2_ by 1 g slag (g),
*CHPC*: Ca(OH)_2_ amount in ordinary mortar (g),
*CHSL*: Amount of Ca(OH)_2_ in slag mortar at the replacement ratio of r (g),
*r*: Replacement ratio of slag

**Figure 6:**
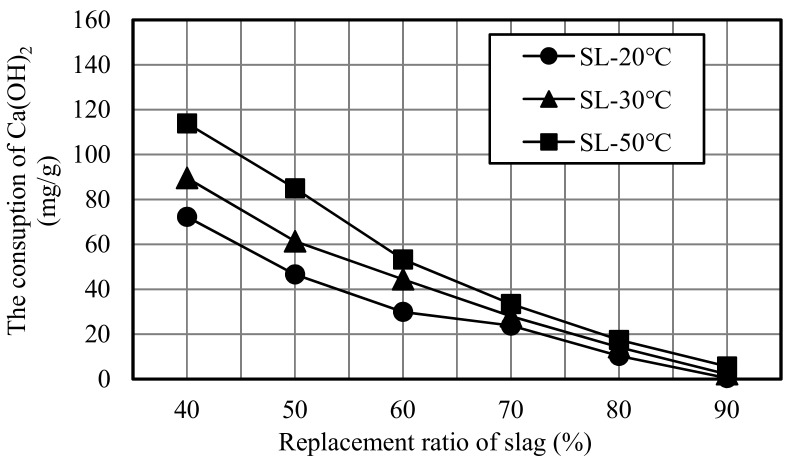
Ca(OH)_2_ consumption of different slag mortars.

From Figure 6, it can be observed that the Ca(OH)_2_ consumption at 50 °C curing temperature was higher than those at 20 and 30 °C curing temperature when the replacement ratio was relatively low, the reaction efficiency of slag obviously increased, causing that the slag unit volume could consume much more Ca(OH)_2_. However, when the replacement ratio was high (exceeded 70%), there was little difference in Ca(OH)_2_ consumption at these three curing temperature. This is also in agreement with Kokubu K, the Ca(OH)_2_ consumption increased obviously when the replacement ratio exceeded 35%.

From what has been discussed above, it can be said that the reaction efficiency of slag can be improved significantly with an increase in curing temperature, but the threshold value of effective replacement ratio of slag in mortar gradually decreased with the increase of curing temperature. The reaction efficiency of slag increased due to the higher curing temperature, and less slag can consume more Ca(OH)_2_ produced by the cement, leading to an increase of the superfluous slag.

### 3.4. Evaluation Method of Reactivity of Slag Mortar

#### 3.4.1. Cement Effective Coefficient (k Value)

The cement effective coefficient (k value) refers to the strength improvement ability of slag in mortar under the condition of different replacement ratios and curing temperatures as well as other parameters such as the mineral admixture or cementitious materials in comparison with the ordinary Portland cement. According to following Equation (2) [27], the cement effective coefficient (k value) can be calculated.

(*C* + *k* × *SL*)/*W* = (*C*/*W*)*eq* (2)

where:

*W*: Unit Water Content (kg/m^3^)
*C*: Unit Cement Content (kg/m^3^)
*k*: Cement effective coefficient
*SL*: Unit slag Content (kg/m^3^)
*(C/W)eq*: Equivalent Water Cement Ratio

Regarding the calculation of *(C/W)eq* value, by formulating the relationship between the water/cement ratio (0.4, 0.5, 0.6) and compressive strengths at 28 days of ordinary Portland cement mortar, and substituting the strength values of different slag mortars at the water/cement ratio of 0.5 into the obtained water/cement ratio-strength Equation, and then the *(C/W)eq* value can be calculated. If the Equation is formed as the replacement ratio (r), the k value also can be calculated according to Equations (3) and (4) as follows.

*r* = *FA*/(*C* + *FA*) (3)

(4) $$k=\left(\frac{\left(C/W\right)eq}{\left(C/W\right)}-1\right)\times \left(\frac{1-r}{r}\right)$$

**Figure 7:**
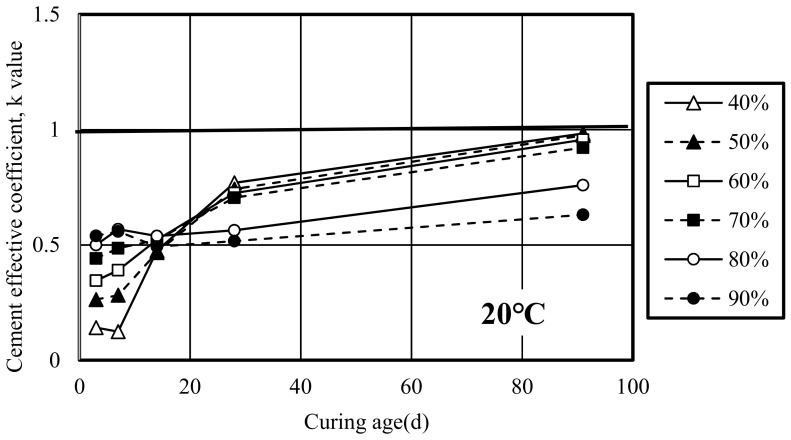

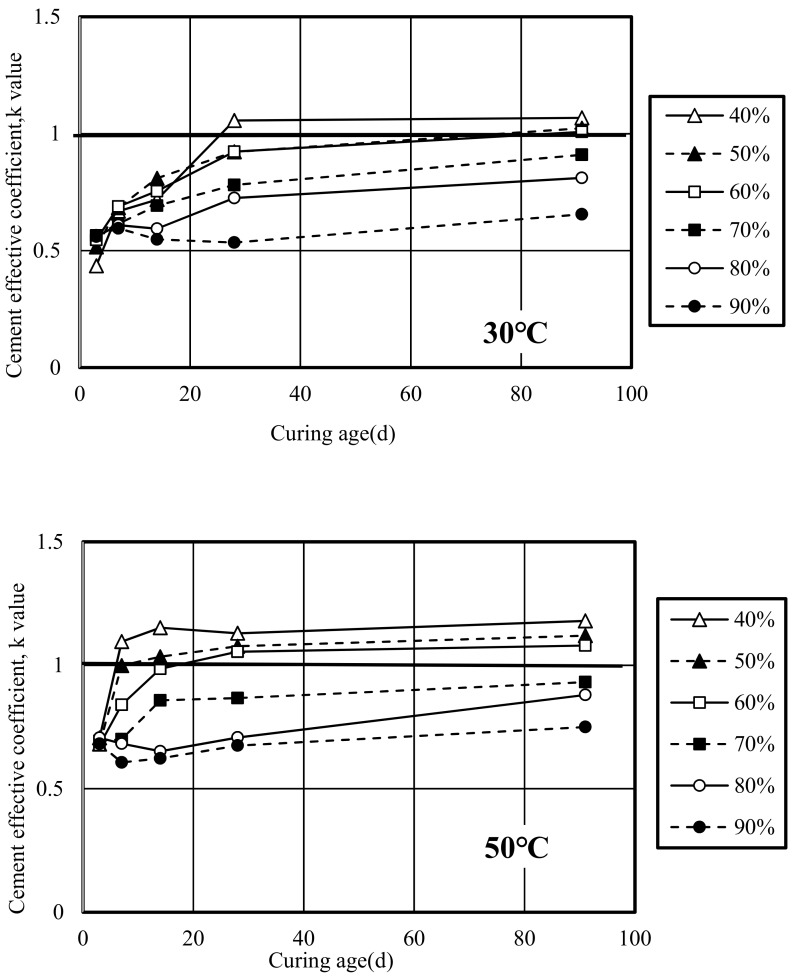
Cement effective coefficient-k value of slag mortar at different curing temperatures.

Based on the above Equation, when the k value > 1, it means that the improvement ability of slag in mortar is higher than ordinary Portland cement, depending on the curing condition and mix proportion; when k value = 1, it reaches the same level as cement; when k value < 1, the improvement ability is worse than that of ordinary Portland cement. The variation curves of the cement effective coefficient (k value) along with the curing age under conditions of different curing temperatures are shown in Figure 7. The k values of different replacement ratios at the early age for three curing temperature cases differed little. At 20 °C, the k values of slag mortar at all replacement ratios at 91 days were lower than 1, which showed that the improvement ability of slag was lower than that of ordinary Portland cement. When the replacement ratio was less than 70%, the k values were mostly close to 1 and peaked at 0.96 at the replacement ratio of 40%. When the replacement ratio exceeded 70%, the k value became very low and decreased obviously at 91 days. In contrast, at 30 °C, at the early age, the k values at each replacement ratio were still lower than 1, at 91 days, the k values at the replacement ratio of 40% and 50% were both higher than 1, and equal to 1 at the replacement ratio of 60%, which showed better or at least the same performance as ordinary Portland cement for strength improvement. When the replacement ratio was over 60%, the k value at the replacement ratio of 70% reached the 0.90. At 50 °C, from 28 days, the k values of slag at the replacement ratio of 40%, 50% and 60% have been higher than 1, at 91 days, the k value at the replacement ratio of 70% was close to 1. The results revealed that the rise of curing temperature could improve the cement effective coefficient of slag, the higher the curing temperature, the higher the reaction efficiency of slag as cementitious material. Through the study of K value, Ogawa found that, curing temperature had a positive effect on improvement of k-value. Even if it is cured in low temperature and fly ash cannot react well in early age, fly ash can react and contribute to strength of mortar as a cementitious material in higher temperature environments [28].The results of slag mortar in this study were similar to those of fly ash mortar in Ogawa’s report.

#### 3.4.2. Relationship between Compressive Strength and Basicity of Mortar

**Figure 8:**
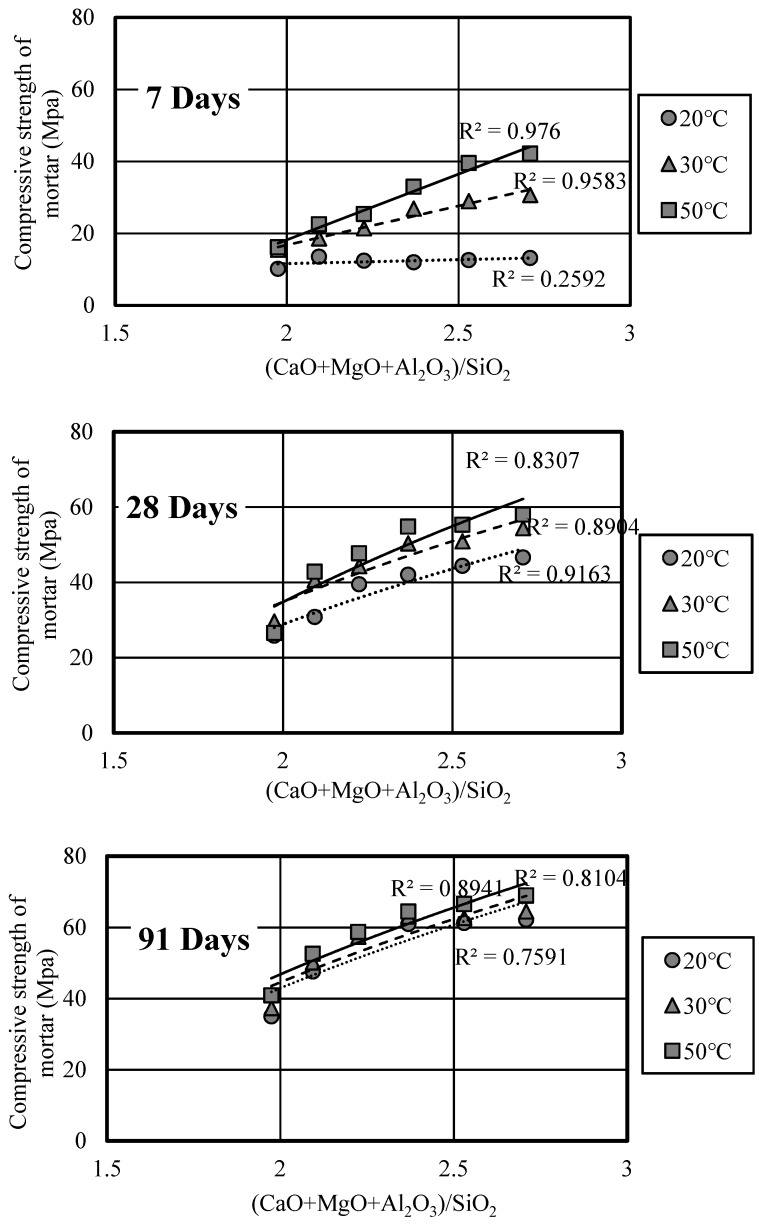
Relationship between the strength and (CaO+MgO+Al_2_O_3_)/SiO_2_ of mortar.

Under normal circumstances, in order to evaluate the reactivity of slag more simply, SiO_2_ is regarded as the major component of the reaction of slag [29]. Therefore, the (CaO + MgO + Al_2_O_3_)/SiO_2_ ratios of basicity of slag mortar (paste) were used to estimate the reaction efficiency of slag in this study. Figure 8 illustrates the relationship between compressive strength and the basicity at different curing ages under conditions of different curing temperatures. It can be observed that the strength of slag mortar at each age all increased with the increase of (CaO + MgO + Al_2_O_3_)/SiO_2_ ratios under the condition of different curing temperatures, the higher (CaO + MgO + Al_2_O_3_)/SiO_2_ ratio, the replacement ratio of slag is lower, leading to a higher compressive strength. Comparing these three curing temperature cases, the approximate curve of 50 °C curing temperature was relatively gentle, which showed the reaction efficiency of slag was higher, leading to a slight difference in strength. Under the conditions of the same (CaO + MgO + Al_2_O_3_)/SiO_2_ content, the compressive strength of slag mortar increased with the curing temperature. Similarly, through the Bogue Equation according to previous studies, the calculation results of (C_2_S + C_3_S)/SiO_2_ were consistent with that of basicity.

#### 3.4.3. Calculation of Reaction Efficiency Coefficient of Slag in Mortar

The first two parts above discussed the reactivity of slag at different curing temperatures qualitatively. Kocaba V. et al. studied five methods to measure the degree of reaction of slag in blended pastes, it showed SEM-BSE-IA-mapping seems to be a promising method to understand and quantify the degree of reaction of slag compared with selective dissolution and differential scanning calorimetry, but the quantitative description was not made in these methods [30,31]. Based on the threshold values of effective replacement ratios of slag at 91 days at different curing temperatures, the threshold value of effective replacement ratio was 70% at 20 °C, which means the amount of Ca(OH)_2_ produced by 30% cement in mortar could be exactly consumed to the lowest value by 70% slag at 20 °C and there was no surplus slag left; the threshold value reduced to 60% at 30 °C and 50% at 50 °C. Along with the increase of the curing temperature, the slag unit volume percentage could react with more Ca(OH)_2_ produced by more ordinary Portland cement, the reaction efficiency of slag increased significantly. As a result, according to the following Equation (5), the reaction efficiency coefficient of slag could be calculated under different curing temperature conditions. For example, at 20 °C, the threshold value (rt) of effective replacement ratio by slag was 70%, the cement amount in the mortar was 30% (1-rt), each 1% slag can exactly react with Ca(OH)_2_ amount produced by (1–70%) /70% cement in mortar at 20 °C, the reaction efficiency coefficient of slag in the mortar was calculated to be 0.42 in this case.

(5) $$E_{T}=\frac{1-r_{t}}{r_{t}}\times 100\%$$

where:

*ET*: Reaction efficiency coefficient of slag at the age of 91 days at (t °C), (%).
$r_{t}$: Threshold value of effective replacement ratio by slag in mortar at the age of 91 days at (t °C), (%).

In contrast with the reaction efficiency coefficient at 20 °C, the reaction efficiency coefficients of slag at 30 and 50 °C were 0.67 and 1, respectively, which means that each 1% slag can exactly react with amount of Ca(OH)_2_ produced by 0.67% and 1% cement in mortar. On this basis, the reaction coefficient evaluation of slag can be more intuitive to reflect the relationship between curing temperature and reaction efficiency. According to the following Equation (6), the reaction efficiency coefficients of slag at different curing temperatures can be obtained. It shows that the curing temperature and reaction efficiency values are linearly correlated, the higher the curing temperature, the reaction activity of slag was stimulated more fully and the contribution of slag on strength of mortar was larger, leading to a higher reaction efficiency coefficient of slag in mortar.

*E* = 0.0189*T* + 0.0657 (6)

where:

*E*: Reaction efficiency coefficient of slag.
*T*: Curing temperature (°C).

In addition, the reaction efficiency of slag at the same temperature will be different depend on curing conditions. In this study, a similar curing condition was tried to achieve as standard curing. From the point of view of complete hydration and curing environment for specimens, the small environment around the specimens under sealed curing condition is very similar to the big environment around the specimens under standard curing condition, therefore, the reaction efficiency of slag under the condition of standard curing in water can be inferred based on the following, Equation (7).

(7) $$E_{WT}=\frac{f_{c}}{f_{cs}}\times E_{T}\times 100\%$$

where:

$E_{WT}$: Reaction efficiency coefficient of slag under the condition of standard curing at (t °C), %.
*ET*: Reaction efficiency coefficient of slag under the condition of sealed curing at (t °C), %.
$f_{c}$: Strength of ordinary cement mortar at 91 days under the condition of standard curing in water (MPa).
$f_{cs}$: Strength of ordinary cement mortar at 91 days under the condition of sealed curing in water (MPa).

From Equation (7), if *fc > fcs*, it can be proven that the standard curing is better than sealed curing, and so the reaction efficiency coefficient of slag under the condition of standard curing increased, leading to *EWT > ET*, and vice versa.

**Figure 9:**
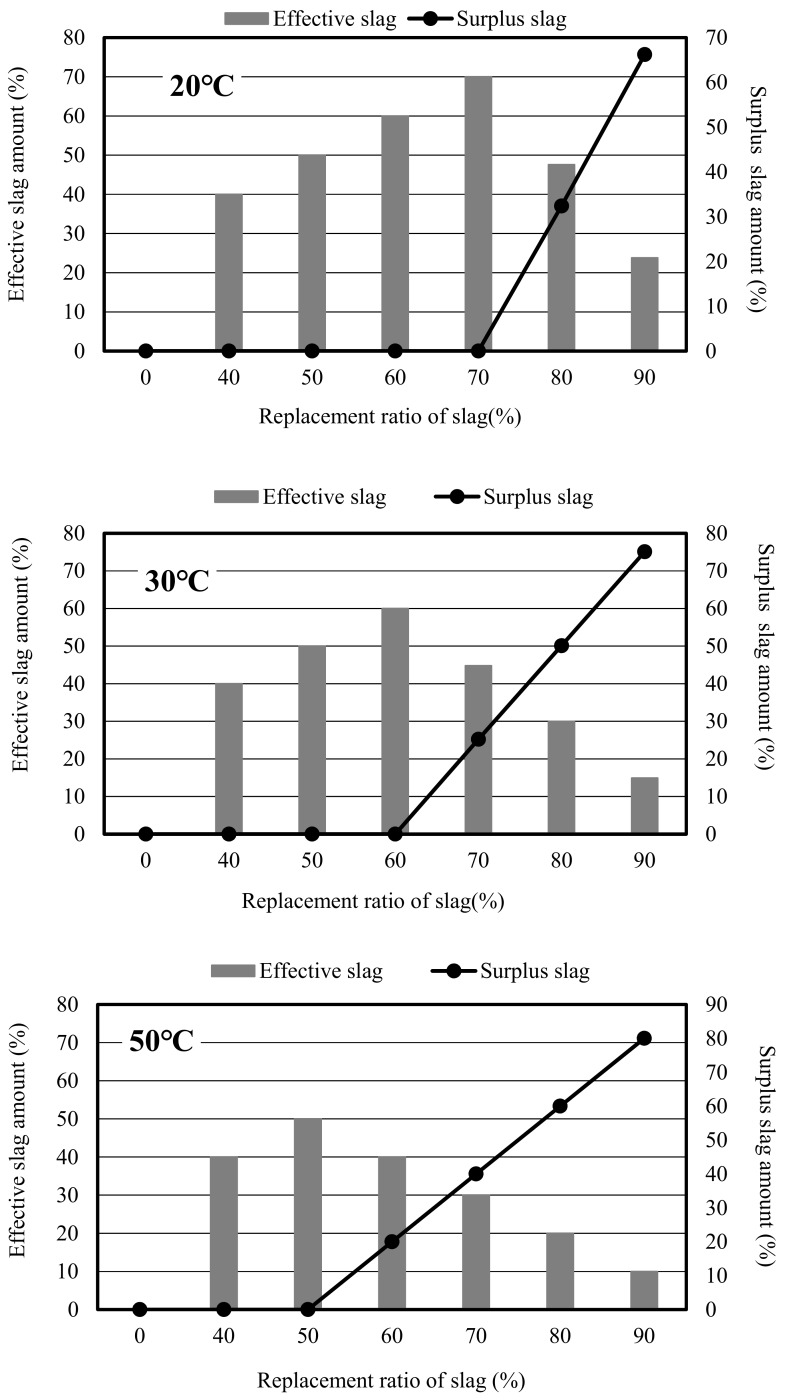
Effective slag amount and surplus slag amount in mortar.

Figure 9 illustrates the relationship between effective (surplus) slag amount and replacement ratio under the condition of different curing temperatures. At the turning point of the surplus slag amount curve, the effective slag amount reached the maximum value; when the surplus slag appeared, the effective slag amount decreased with the increase of the replacement ratio. The curves at the different curing temperatures showed normal distribution and skew normal distribution, the effective slag amounts on both sides of turning point were similar, and a very high surplus of slag led to a low strength.

## 4. Conclusions

The following results were obtained in this study:

(1) The upper limit of effective replacement ratio of GBFS in high volume slag mortar depends on the reaction efficiency of GBFS at different curing temperatures. The reaction efficiency of slag in mortar increased with the increase in curing temperature at 20, 30, and 50 °C, respectively; but the threshold value of the effective replacement ratio of slag decreased with the rise of curing temperature in a contrary fashion. The redundant and ineffective slag has emerged at low replacement ratio at higher temperature.

(2) Based up on the results of strength and Ca(OH)_2_ amount of different slag mortars, the upper limits of effective replacement ratio of slag at 20,30 and 50 °C curing temperature are 70%, 60% and 50%, respectively. When replacement ratio of slag is lower than the upper limit at each temperature, there is almost no the loss in strength compared with ordinary cement mortar.

(3) At low temperature (20 and 30 ℃), due to the low reaction efficiency of GBFS in mortar at the early age, the strength decreases linearly with replacement ratio until 91 days. In contrast, at higher temperature of 50 ℃, the reaction efficiency of slag and the hydration process of cement can be improved, and the activity of slag can be stimulated to consume the Ca(OH)_2_ at early curing age. The obvious turning point of strength curve was found at 7 days. Since a large amount of effective slag has been stimulated to participate in the reaction at early curing age, the strength development of mortar slows down at the longer curing time due to the existence of ineffective slag.

(4) At 20 °C curing temperature, the k values of slag mortar at each replacement ratio at 91 days were all lower than unity, which showed that the strength improvement ability of slag was worse than that of ordinary Portland cement. In contrast, at 30 and 50 °C curing temperature, the k values exceeded unity at 91 days when the replacement ratio of slag in mortar was below 70%, and the strength improvement ability of slag was obviously enhanced.

(5) Through the derivation of an empirical formula, the reaction efficiency coefficient of slag was calculated as 0.42 at 20 °C, which means that each 1% slag can exactly react with the amount of Ca(OH)2 produced by 0.42% cement in mortar at 20 °C. In contrast, the reaction efficiency coefficients of slag were improved at 30 °C and 50 °C to 0.67 and 1, respectively. On this basis, the relationship Equation between the curing temperature and reaction efficiency coefficient is established, the reaction efficiency coefficient and upper limit of effective replacement ratio of slag at different temperatures can be calculated directly in a practical engineering application. In addition, the conversion formula for the reaction efficiency coefficient of slag under standard curing conditions was also determined, which provided a certain reference significance for the calculation of reaction efficiency coefficient of slag under different curing conditions in practical construction.

(6) Thermal curing can accelerate the early strength development of high-volume slag mortar or concrete. Although the later strength development slowed down, the strength was still higher than that of concrete at low temperature, which can meet the construction requirements. This result can provide a reference for rapid construction and emergency repair projects.

(7) Due to the conditions limitation, this paper aims to study the upper limits of effective replacement rate of GBFS under different curing temperatures, the relationship between curing temperature and reaction efficiency coefficient of GBFS is clarified, a calculating formula for reaction efficiency coefficient-temperature of GBFS is established, and a conversion formula of the reaction coefficient of GBFS under different curing conditions is put forward. Thus, the reaction efficiency coefficients under different curing conditions such as standard curing or curing in air can both be calculated, which provides a theoretical basis for practical construction. Regarding how engineers are going to cure the samples and at the same time keep them under water and curing at elevated temperatures, they can be continuously heated by a circulating heating pump or heater to keep the temperature high and stable.

## Figures and Tables

**Table 1:** Physical properties of ordinary Portland cement.

Density (g/cm^3^)	Specific Surface Area (cm^2^/g)	Setting Time			Compressive Strength (N/mm^2^)		
		Water (%)	Initial (h-m)	Final (h-m)	3d	7d	28d
3.16	3350	28.8	2-00	3-12	27.0	45.4	65.0

**Table 2:** Chemical composition of cement and slag.

Type	Ig.Loss (%)	SiO_2_	Al_2_O_3_	Fe_2_O_3_	CaO	MgO	SO_3_	Na_2_O	K_2_O	TiO_2_
Cement	0.7	19.54	4.68	3.17	65.66	1.77	2.99	0.28	0.57	0.28
Slag	0.1	34.11	14.56	0.28	43.51	5.54	--	0.24	0.34	0.54

**Table 3:** Mix proportion design of different slag mortar.

Mineral Admixture	W/(SL + C) (%)	Replacement Ratio SL/(SL + C) (%)	Unit Content (kg/m^3^)			
			W	C	SL	S
Blast furnace slag	50	0	306	612	0	1310
		40	304	365	243	1310
		50	302	302	302	1310
		60	300	240	360	1310
		70	298	179	417	1310
		80	298	119	476	1310
		90	295	59	531	1310
Cement	40	0	279	698	0	1310
	60	0	327	546	0	1310

W: water; C: cement; SL: slag; S: natural sand).
